# Efficacy of Sodium-Glucose 2 Transporter Inhibitors in Heart Failure With Preserved Ejection Fraction: A Narrative Review

**DOI:** 10.7759/cureus.69623

**Published:** 2024-09-17

**Authors:** Rafik Maged, Mohit Sinha, Hema Manvi Koneru, Hooria Sarwar, Venkata Varshitha Bandi, Pakeeza Tarar, Nouran Halawa

**Affiliations:** 1 Internal Medicine, California Institute of Behavioral Neurosciences and Psychology, Fairfield, USA; 2 Psychiatry, California Institute of Behavioral Neurosciences and Psychology, Fairfield, USA

**Keywords:** diastolic dysfunction, diastolic heart failure, empagliflozin, hfpef, sodium-glucose cotransporter-2 (sglt2) inhibitors

## Abstract

Heart failure with preserved ejection fraction (HFpEF) is an increasingly prevalent condition. It occurs more commonly in older patient populations with multiple comorbidities, such as hypertension, diabetes, and obesity. However, managing HFpEF has been challenging due to its complex pathophysiology, and medications effective for heart failure with reduced ejection fraction (HFrEF) have not shown similar efficacy in HFpEF. Sodium-glucose 2 transporter (SGLT2) inhibitors were originally developed for the treatment of type 2 diabetes mellitus, yet several trials and papers have proved their significant role in HFpEF. Through a variety of mechanisms, including natriuresis, diuresis, and anti-inflammatory effects, to name a few, this class of drugs has shown promising results in HFpEF patients. The use of SGLT2 inhibitors in HFpEF has resulted in improvements in several aspects, including biomarkers, imaging, symptomatology, and mortality. Moreover, SGLT2 inhibitors have a favorable safety profile, which is especially significant given the high comorbidity burden in HFpEF patients. This feature is particularly notable given the type of patient being managed. Extensive research is still being undertaken for their use in HFpEF, given the positive results obtained thus far.

## Introduction and background

According to the American Heart Association (AHA) and the European Society of Cardiology (ESC), the prevalence of heart failure with preserved ejection fraction (HFpEF) is on the rise, with studies suggesting that it is responsible for almost 50% of the cases of heart failure (HF) in the United States. HFpEF is increasingly recognized as a major public health concern due to its significant impact on morbidity and mortality, especially in aging populations. It is also more likely to occur in patients with comorbidities, such as hypertension, diabetes, and obesity [1]. This noticeable surge in the prevalence of HFpEF is partially attributed to the increased survival rates among patients with other cardiovascular diseases, which naturally leads to more patients developing HF as they age [2].

HF is a cardiac condition that is associated with reduced cardiac output (CO) and/or increased intraventricular pressures, which can result in a set of clinical signs (elevated jugular venous pressure (JVP), pulmonary crackling, and peripheral edema) and symptoms (dyspnea and fatigue) [3]. In clinical practice, HF is categorized into three distinct types based on ejection fraction (EF): heart failure with reduced ejection fraction (HFrEF), defined as HF with EF < 40%; heart failure with mid-range ejection fraction (HFmEF), characterized by EF ranging from 40% to 49%; and HFpEF, identified in patients with EF ≥ 50% [3].

Although HF is commonly associated with impairment in systolic function, a significant portion of patients with signs and symptoms of HF have HFpEF. In such patients, diastolic dysfunction is the primary culprit. The syndrome whereby a patient has manifestations of HFpEF and no valvular abnormalities is referred to as diastolic heart failure (DHF) [4]. During diastole or myocardial relaxation, the ventricles fill with blood. For this to occur adequately, a pressure gradient is created between the atria and the ventricles, along which blood flows into the ventricles. In HFpEF, the myocardium is stiff, which results in a reduced pressure gradient and inevitably leads to impaired ventricular filling. Since the ventricles are not properly filled, less blood will be pumped out during systole; yet, the EF will remain unchanged. The diagnosis of diastolic dysfunction is, doubtlessly, a major challenge because of the overlap of the symptomatology of DHF with other diseases, such as obesity and pulmonary disorders [5]. Additionally, the diagnosis of HF is mostly clinical, which, on its own, is subject to fallacy.

Several modalities in the management of HFrEF have been established. These approaches include targeting symptom control, limiting disease progression, and enhancing quality of life. They include, but are not limited to, lifestyle modification (including exercise and weight loss), pharmacological agents (e.g., diuretics and beta-blockers (BBs)), and management of associated comorbidities such as sleep apnea [6]. Although neurohumoral inhibitors, such as angiotensin-converting enzyme inhibitors (ACEis), mineralocorticoid receptor antagonists (MRAs), and BBs, have long been proven to reduce mortality and hospitalization in HFrEF, there remains uncertainty about their effect on HFpEF patients [3]. Therefore, it is necessary to explore alternative drug classes that might benefit HFpEF patients in this regard.

Sodium-glucose 2 transporter (SGLT2) inhibitors are a renowned class of medication developed initially as a line of treatment for type 2 diabetes mellitus (T2DM). Their primary site of action is the kidney, specifically the proximal convoluted tubule (PCT), where they inhibit glucose reabsorption into the bloodstream, contributing to lower blood glucose levels, which is integral for diabetic patients.

In 2017, SGLT2 inhibitors were first explored for their potential use in HFpEF in the EMPEROR-PRESERVED trial (Empagliflozin Outcome Trial in Patients With Chronic Heart Failure With Preserved Ejection Fraction). This trial showed promising results that support their use for reducing mortality and hospitalization rates, as well as facilitating symptom control [7].

Currently, there are numerous challenges that face physicians and scientists regarding HFpEF. Firstly, the pathophysiology is not fully understood, making the task of developing targeted therapies cumbersome. Secondly, more research is still required to establish consensus on the management of HFpEF. Thirdly, the complex nature of the patients suffering from HFpEF, due to the presence of multiple comorbidities, makes administering and experimenting with pharmacological interventions an intricate task. Finally, the lack of sufficient data on reliable markers in the diagnosis and risk stratification of HFpEF leads to difficulty in the objective assessment of the efficacy of treatments used.

In this article, you will find a concise overview of SGLT2 inhibitor use for the management of HFpEF, including the theories that try to explain its mechanism of action and potential benefit, as well as the clinical significance of its use, among other controversial data regarding its impact.

## Review

Mechanism of action of SGLT2 inhibitors

The PCT of the kidney reabsorbs around 90% of the filtered glucose, along with sodium, using the SGLT2 proteins. Therefore, SGLT2 inhibitors were specifically developed to target the SGLT2 proteins and lower blood glucose levels in diabetic patients. As a result, the normal renal threshold for glucosuria of 180 mg/dL decreases [8]. An advantage of SGLT2 inhibitors, compared to other drugs, is the significantly lower risk of hypoglycemia in both diabetic and non-diabetic patients [8].

Alteration of some homeostatic processes due to the comorbidities that usually accompany HFpEF [9] results in up-regulation of SGLT2 proteins, which increases reabsorption of sodium in the PCT and causes fluid overload and suboptimal response to diuretics, highlighting the potential benefit of using SGLT2 inhibitors [10].

HFpEF is a complex disease involving several comorbidities that contribute to the production of a sustained, low-grade pro-inflammatory process, which increases oxidative stress. This gives rise to cardiac remodeling and, in turn, diastolic dysfunction, which is a major player in HFpEF, as mentioned previously [9]. Several studies have highlighted the antioxidative effects of SGLT2 inhibitors on the heart. Interestingly, treatment with empagliflozin, an SGLT2 inhibitor, significantly reduced oxidative stress markers such as glutathione and 3-nitrotyrosine, as well as inflammatory markers including tumor necrosis factor-alpha and IL-6, in cardiomyocytes isolated from HFpEF patients studied in vitro [11]. Inhibition of calcium and calmodulin-dependent protein kinase II may contribute to the amelioration of oxidative stress and inhibition of inflammatory mechanisms, such as the NOD-like receptor protein 3 (NLRP3) inflammasome [12]. This is particularly notable because this low-grade pro-inflammatory state, often observed in HFpEF, can contribute to disease progression and worsening symptoms [11].

Experiments with empagliflozin have shown that it enhances left ventricular (LV) diastolic function by promoting phosphorylation of cardiomyocyte myofibers in both human and rat myocardium [13]. Another proposed mechanism, shown in recent research in rodent and human models of HFrEF and HFpEF, is that empagliflozin treatment enhances diastolic function by reducing passive myofilament stiffness, attributed to increased titin phosphorylation [11,13]. These factors could be key in the SGLT2 inhibitor-mediated improvement of diastolic dysfunction. Other proposed mechanisms involved in the improvement of diastolic function in patients with HFpEF include decreased arterial stiffness [14] and a shift of energy consumption to ketone oxidation [15].

SGLT2 inhibitors benefit HF patients (HFrEF, HFmrEF, and HFpEF) most probably due to diuresis, improved cardiomyocyte metabolism, increased erythropoietin (EPO) release, and modifications in cardiac ion channels. SGLT2 inhibitors have been shown to not only inhibit cardiac remodeling but also contribute to its regression, regardless of HF status or diabetic status [16].

Clinical significance of the use of SGLT2 inhibitors

SGLT2 inhibitors are particularly useful for HFpEF patients because they exhibit positive effects on obesity, hypertension, and diabetes [17]. Firstly, the excretion of glucose caused by SGLT2 inhibitors has been shown to result in a weight reduction of about 2 to 3 kg [18]. By promoting natriuresis and diuresis, SGLT2 inhibitors can significantly reduce the preload and afterload of the heart [19]. The use of SGLT2 inhibitors has also been associated with reductions in blood pressure by 3 to 5 mmHg. Importantly, this reduction in blood pressure is not accompanied by reflex tachycardia and increased sympathetic drive [20].

One study found that empagliflozin treatment led to a significant reduction in left ventricular mass index (LVMi), which is a marker of LV hypertrophy (often seen in HFpEF patients) [21]. Another study reported that canagliflozin significantly reduced E/e', an echocardiographic parameter of diastolic dysfunction [22]. A study involving diabetic patients showed that six months of dapagliflozin treatment significantly improved both E/e' and LVMi [23]. Additionally, canagliflozin significantly reduced N-terminal pro-B-type natriuretic peptide (NT-proBNP) levels, a biomarker of HF, over two years [24]. Although it is challenging to draw definitive conclusions, it can be inferred that SGLT2 inhibitors tend to improve LVMi and E/e' (by reducing both) [25].

A small study found that six months of dapagliflozin therapy significantly improved LV diastolic function (measured by E/e′ and left atrial volume index) in HFpEF patients with diabetes, despite no significant change in brain natriuretic peptide (BNP) levels [26]. Further evaluation showed that this therapy also significantly improved LV longitudinal strain in HFpEF patients, but not in HFrEF patients [26]. This improvement in LV longitudinal strain was independent of demographic, clinical, and echocardiographic parameters related to E/e′ after dapagliflozin treatment. Another study on the effects of canagliflozin on LV remodeling in diabetic patients with HF demonstrated a significant reduction in LV filling pressure (assessed by E/e′) after six months of treatment, with this effect lasting even after 12 months of therapy [27].

There are potential reasons why one study did not show improvement in E/e' or e' with ipragliflozin. One of these reasons was an intervention period of 24 weeks, which may have been insufficient [28]. Sitagliptin showed a reduction in E/e' after a two-year follow-up period [29]. Therefore, longer-term studies are necessary to assess the impact on LV diastolic function [25].

One study demonstrated that, in a group of 53 T2DM patients with HF (including HFrEF, HFmrEF, and HFpEF), dapagliflozin improved systolic and diastolic LV function, assessed by several echocardiographic parameters such as global longitudinal strain and E/e′ [27]. Improved diastolic function was also noted in diabetics participating in smaller trials with empagliflozin or canagliflozin [21]. Additionally, one study showed that empagliflozin enhanced diastolic function in diabetic patients, while parameters such as stroke volume index, cardiac index, and vascular resistance index remained the same [30].

One study was a randomized controlled multicenter trial examining the effects of ipragliflozin in older T2DM patients with HFpEF. The findings indicated that ipragliflozin did not improve the primary endpoint of LV diastolic function or secondary endpoints, such as echocardiographic parameters, plasma NT-proBNP levels, New York Heart Association (NYHA) classification, HbA1c levels, and blood pressure, compared to conventional treatment [25]. However, subgroup analysis showed a reduction in LVMi in participants aged 70 years and older and a decrease in NT-proBNP levels in participants with baseline NT-proBNP of 400 pg/mL or higher. This suggests that ipragliflozin might help reduce LV hypertrophy or lower NT-proBNP [25].

Impact on symptoms and functional limitations

Although recently conducted trials have shown some evidence regarding reduced rates of hospitalization and cardiovascular death (CVD) in patients with HFpEF treated with SGLT2 inhibitors, there still remains a lack of evidence regarding their effect on quality of life and functional capacity. In HFrEF, many drug therapies, such as renin-angiotensin receptor blockers, MRAs, and angiotensin receptor-neprilysin inhibitors, have been shown to significantly improve patients’ lives and their functional capacity [31]. However, the aforementioned benefits were not observed in HFpEF patients receiving those medications [32], where other factors, like obesity and hypertension, play a major role [33].

The effect of SGLT2 inhibitors on functional limitation and quality of life was measured using the Kansas City Cardiomyopathy Questionnaire (KCCQ). The KCCQ is a questionnaire consisting of 23 items, which is self-administered by patients with HF to assess quality of life, functional limitation, social limitation, and symptom burden. A total of four scores are calculated: the total symptom score (TSS), the clinical symptom score (CSS), the physical limitation score (PLS), and the overall summary score (OSS) [34].

In a combined analysis of the DELIVER (Dapagliflozin Evaluation to Improve the Lives of Patients With Preserved Ejection Fraction Heart Failure) and DAPA-HF (Dapagliflozin in Patients With Heart Failure and Reduced Ejection Fraction) trials, dapagliflozin markedly enhanced the KCCQ-TSS at both four months and eight months, compared to placebo. This improvement was observed across various KCCQ domains and most key subgroups [34]. Notably, at eight months, the improvement in KCCQ-TSS was more pronounced in participants with T2DM, compared to those without. Overall, dapagliflozin led to better health status outcomes across all KCCQ domains and was effective regardless of EF, including in individuals with a left ventricular ejection fraction (LVEF) above 60% [34].

A recent analysis of the DELIVER trial indicates that patients with HFpEF who have more severe symptoms at the start may see greater improvement with dapagliflozin compared to placebo. However, this conclusion is not definitive, and other factors might explain the differences observed [35]. In the PRESERVED-HF trial (Effects of Dapagliflozin on Biomarkers, Symptoms and Functional Status in Patients With Preserved Ejection Fraction Heart Failure), which compared dapagliflozin with placebo in patients with HFpEF, those on dapagliflozin showed a significant and meaningful improvement in their six-minute walk test (6MWT) distance, compared to those receiving a placebo. The analysis of this planned secondary endpoint found that patients on dapagliflozin were 66% more likely to achieve at least a 15-meter improvement in 6MWT distance over 12 weeks than those on the placebo [36].

While the EMPEROR-PRESERVED trial suggested a possible decrease in benefit regarding symptomatic and functional improvement at the highest EF levels [37], the analysis of the DELIVER trial showed no evidence of variation related to LVEF. The treatment effects were consistent among patients with an LVEF of 60% or higher and those with less than 60%. This indicates that the beneficial effects of SGLT2 inhibitors are evident regardless of EF [38]. This discrepancy might be due to the more severe baseline conditions in the DELIVER trial [35].

Hospitalization, CVD, and mortality

In patients with HFmrEF and HFpEF, SGLT2 inhibitors reduced the rates of hospitalization for HF and CVD. Regarding CVD and all-cause mortality as separate entities, there was no significant reduction by SGLT2 inhibitors, indicating that their main benefit lies in decreasing HF hospitalizations in these patients. The precise mechanism behind this effect remains unclear, but multiple hypotheses have been suggested. HFpEF patients often have hypertension, hyperglycemia, and obesity, which increase the risk of morbidity and mortality [39], but these conditions can be positively impacted by SGLT2 inhibitors, potentially benefiting those patients [40].

According to one study, the empagliflozin group had a lower number of overall (both initial and recurrent) hospitalizations for HF and cardiovascular causes, in comparison to patients who received a placebo [41]. Empagliflozin decreased the likelihood of hospitalization for HF. The hospitalizations that were prevented ranged from those managed with oral or intravenous diuretics to those necessitating intravenous vasopressors, positive inotropic agents, or intensive care [41]. Empagliflozin also had a significant impact on outpatient HF management, reducing the frequency of worsening HF events. Patients treated with empagliflozin had less frequent encounters at the emergency department for HF exacerbations than those who received a placebo. The use of SGLT2 inhibitors reduced visits necessitating a dosage increase of diuretics for worsening HF and increased the number of visits reporting a reduction in diuretic dose. Furthermore, patients receiving empagliflozin were more likely to have an improved NYHA class. The aforementioned improvements were noted in the initial phase and continued throughout the trial [41].

According to data primarily derived from the SOLOIST-WHF trial (SGLT2 Inhibition With Dapagliflozin in Patients With Heart Failure and a Reduced Ejection Fraction), which was the leading study in terms of hard evidence regarding the benefits of SGLT2 inhibitors in HFpEF with DM [42], a meta-analysis concluded that SGLT2 inhibitors are associated with a greater likelihood of mitigating the risk of cardiovascular disease and hospitalization for HF in patients with HFpEF [43].

A meta-analysis of trials in the HFrEF population showed that SGLT2 inhibition caused a reduction in the risk of CVD or hospitalization for HF [44]. This systematic review and meta-analysis demonstrated that SGLT2 inhibitors provided similar benefits in reducing HF hospitalization or CVD in patients with HFmrEF and HFpEF, primarily driven by their impact on hospitalization for HF [45]. Unlike previous meta-analyses of HFmrEF and HFpEF populations, this study's sensitivity analysis indicated a trend toward significant effects of SGLT2 inhibitors in preventing CVD. The hazard ratio for CVD in HFmrEF and HFpEF was similar to that found in HFrEF [44].

One study concluded that SGLT2 inhibitors are associated with a decreased risk of HF hospitalization and mortality in HF patients compared to placebo, regardless of diabetes status, the specific SGLT2 inhibitors used, or the length of follow-up. In patients with HFpEF, the use of SGLT2 inhibitors showed a tendency to benefit in terms of reducing the outcomes of CVD [46].

While several studies identified that all-cause mortality and cardiovascular mortality were comparable between patients on SGLT2 inhibitors and those on placebo [42], one study reported a significant reduction in all-cause mortality in HF patients (both HFpEF and HFrEF) treated with SGLT2 inhibitors [47].

Adverse effects and safety profile

SGLT2 inhibitors are generally well tolerated; however, they are associated with significant adverse effects, such as urinary tract infection (UTI) due to increased glucose excretion in the urine [48]. Additionally, owing to their diuretic effect, hypovolemia and hypotension can occur. They have also been implicated in euglycemic ketoacidosis, diabetic ketoacidosis (DKA), and acute kidney injury (AKI) [10].

The findings of one meta-analysis, summarizing the safety outcomes of SGLT2 inhibitors in patients with HFpEF and HFmrEF, indicated that SGLT2 inhibitors had a favorable safety profile without an increase in major adverse effects. This is despite the fact that the data acquisition methods and definitions of adverse events (AEs) varied across the different trials included in the meta-analysis [47].

Regarding the safety profile of SGLT2 inhibitors, AEs were more likely to occur with increasing age. For patients receiving dapagliflozin, in relation to those on placebo, pre-specified safety outcomes were comparable regardless of age, including serious AEs.

Discontinuation rates were similar between patients on dapagliflozin versus patients on placebo, irrespective of age [49]. Although patients of older age (65 years or older) are at an increased risk for renal complications, the incidence of major renal AEs was similar between those treated with dapagliflozin and those given placebo, even among those aged 75 and older. The use of dapagliflozin was not associated with hypovolemia at higher rates in comparison with other medications with diuretic effects, particularly in the older population. In addition, the risk of hypoglycemic events and DKA was not significantly increased with dapagliflozin use. This indicates that dapagliflozin is safe for use across all age groups in patients with HFmrEF and HFpEF [49].

The EMPULSE trial (Empagliflozin in Patients Hospitalized With Acute Heart Failure Who Have Been Stabilized) included subjects with an EF above 40%, indicating that it is both safe and effective to begin empagliflozin treatment in a hospital setting [50]. Similarly, dapagliflozin has shown benefits for these patients, particularly in improving symptoms and exercise capacity. Guidelines issued by major cardiovascular professional societies, including the AHA, among others, recommend SGLT2 inhibitors with a class 'IIa' designation for patients with HFpEF, as the benefits are likely to outweigh the risks, provided there are no contraindications [50].

Limitations

This study has a few potential limitations. Firstly, there was limited access to literature, which led to a limited number of papers being included in this review. Secondly, we included only English papers, which may have led to language bias. Thirdly, existing data and literature regarding the long-term effects of SGLT2 inhibitors on patients with HFpEF were limited, as most of the trials conducted had a relatively short follow-up period. Fourthly, the differences in ethnicities, gender, and age have not been addressed in detail. The margin for improvement is wide and comprises access to a more inclusive pool of articles, further research into the effects of SGLT2 inhibitors with longer follow-up periods, and a more comprehensive assessment of patient characteristics.

Future considerations

Further investigations and trials with longer follow-up periods are required to explore the long-term effects of SGLT2 inhibitors on HFpEF patients. In addition, more research is needed to address disparities across different demographics, such as age, gender, and ethnicity. Additionally, research discussing patient-centered outcomes and quality of life needs to be explored.

## Conclusions

The use of SGLT2 inhibitors in patients with HFpEF has shown positive outcomes. Studies like EMPEROR-PRESERVED and DELIVER have found that these drugs can help reduce hospital visits, alleviate symptoms, and improve quality of life. In addition, they are likely to be effective in improving the diastolic function of the heart, which can prove to be an immense accomplishment for the management of patients with HFpEF. With HFpEF becoming more prevalent, it is apparent that more personalized treatment options are needed. This condition is complex and can greatly differ from one patient to another, which makes finding a universal treatment plan difficult. This is because multiple factors, including comorbidities, age, and weight, influence the pathophysiology of HFpEF and have a significant impact on the way medical treatment takes effect. It is also crucial to consider how treatments affect people’s overall quality of life and daily function. Non-drug approaches, such as exercise and dietary changes, are worth exploring as well, as they could complement medications and offer additional benefits.
